# Constrictor responses of cerebral resistance arterioles in male and female rats exposed to prenatal alcohol

**DOI:** 10.14814/phy2.15079

**Published:** 2021-10-29

**Authors:** Partha S. Saha, Tiffany M. Knecht, Denise M. Arrick, Michael J. Watt, Jamie L. Scholl, William G. Mayhan

**Affiliations:** ^1^ Division of Basic Biomedical Sciences Sanford School of Medicine University of South Dakota Vermillion South Dakota USA

**Keywords:** cerebral ischemia, fetal alcohol syndrome, stroke, vasoconstriction

## Abstract

While it is known that dilation of cerebral arterioles to NOS‐dependent agonists is impaired in rats exposed to prenatal alcohol, no studies have examined the influence of prenatal alcohol on constrictor response of cerebral arterioles. Our goal was to determine whether constrictor responses of cerebral resistance arterioles are altered by prenatal exposure to alcohol and if any changes differed as a function of age or sex. We fed Sprague‐Dawley rat dams a liquid diet with or without alcohol (3% ethanol) for the duration of their pregnancy. We then examined reactivity of cerebral arterioles to thromboxane (U‐46619; 0.01 and 0.1 µM), arginine vasopressin (0.1 and 1 nM), and angiotensin II (1 and 10 µM) in four groups of offspring: control male and female, and prenatal alcohol male and female at two different ages (adolescent: 4–6 weeks old and adult: 14–16 weeks old). Constriction of cerebral arterioles to U‐46619 and AVP were similar in male and female rats regardless of exposure to prenatal alcohol and age. Similarly, adolescent male and female rats showed no difference to angiotensin II following prenatal exposure to alcohol. However, alcohol‐exposed females exhibited an unexpected dilation to the high concentration of angiotensin II in adulthood, which was absent in males. We suggest that the findings from these studies may have implications regarding the susceptibility of the brain to cerebral ischemic damage. We speculate that impaired vasodilation, coupled with preserved vasoconstriction, can lead to a scenario favoring a decrease in cerebral blood flow during times of increased metabolic demand.

## Graphical abstract

Our goal was to examine constrictor responses of cerebral arterioles in rats exposed to prenatal alcohol. We found that constrictor responses were preserved in adolescent and adult rats that were exposed to prenatal alcohol. We suggest that impaired dilation, with preservation of constriction, may favor a scenario leading to cerebral ischemia, and perhaps stroke.

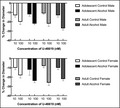

## 1 INTRODUCTION

Although alcohol is a well‐known teratogen, about 10% of women in the United States admit consuming alcohol during their pregnancies (Tan et al., 2015). Since the development of the central nervous system occurs for an extended period of time, exposure to alcohol at any time during development has the likelihood to produce significant damage to all components of the central nervous system. Individuals diagnosed with fetal alcohol spectrum disorder (FASD) exhibit a wide range of behavioral, cognitive, physical, and neurological impairments during childhood, adolescence, and adulthood (Jones, 2011; Riley et al., 2011).

We have suggested that abnormalities associated with FASD may be related to alterations in reactivity of cerebral arterioles (Cananzi & Mayhan, 2019a, 2019b). Our previous studies have shown that endothelial nitric oxide synthase (eNOS)‐ and neuronal nitric oxide synthase (nNOS)‐dependent responses of cerebral arterioles in adolescent and adult rats are blunted by prenatal exposure to alcohol (Cananzi & Mayhan, 2019a, 2019b; Saha et al., 2021). This diminished vasodilation would lead to a mismatch in cerebral blood flow during periods of increased metabolic demand, that is, impairment in neurovascular coupling. It is also possible that alterations in vasoconstrictor responses of cerebral arterioles by prenatal exposure to alcohol would be detrimental. We suggest that preservation or exaggerated vasoconstriction, coupled with impaired vasodilation, would favor a scenario that would contribute to cerebral ischemia during appropriate conditions. While one study has shown that contraction of the aorta to norepinephrine is reduced, while contraction to potassium chloride is intact, in rats exposed to prenatal alcohol (Turcotte et al., 2002), no studies to our knowledge have examined constrictor responses of cerebral arterioles in animals exposed to prenatal alcohol. Further, there are few studies that have examined whether responses of cerebral arterioles following prenatal exposure to alcohol differ as a function of sex or age. Thus, the goal of the current study was to examine the in vivo responses of cerebral resistance arterioles to important vasoconstrictors in male and female rats at different age groups following exposure to prenatal alcohol.

## 2 MATERIALS AND METHODS

All procedures were reviewed and approved by the Institutional Animal Care and Use Committee at the University of South Dakota and were performed in accordance with the National Institutes of Health Guide for the Care and Use of Laboratory Animals.

### 2.1 Experimental diets

We used virgin adult male and female Sprague‐Dawley rats for breeding. One male and one female rat were allowed to mate over a period of 7 days. Dams were then housed singly and assigned randomly to groups that were fed one of the following liquid diets for the entire gestation period (21–23 days): control (0% alcohol) diet or 3% alcohol diet. Liquid diets were prepared daily, as we have described previously (Cananzi & Mayhan, 2019a, 2019b; Saha et al., 2021). The control diets contained 1.0 kcal/ml of which 35% are derived from fat, 47% from carbohydrates, and 18% from protein. The 3% alcohol diets contained 1.0 kcal/ml of which 35% are derived from fat, 18% from protein, 29% from carbohydrate, and 18% from alcohol. The total daily volume of diet fed to the control animals was based upon the daily consumption of diet by the alcohol animals. The alcohol diet results in a blood alcohol concentration (BAC) equivalent to a BAC of 0.06% (Cananzi & Mayhan, 2019a). The liquid diets fed to the dams were replaced by a normal chow/water diet within a day of the birth of the pups. Rats were cross‐fostered and were weaned at 3 weeks of age and placed in cages with those of the same sex. Both male and female rats were used for these experiments.

### 2.2 Experimental protocol

The rats were prepared for studies at 4–6 and 14–16 weeks of age and were obtained from multiple different litters. On the day of the experiment, the rats were anesthetized with thiobutabarbital sodium (Inactin, 100 mg/kg IP) and a tracheotomy was performed. The rats were ventilated mechanically with room air and supplemental oxygen. A catheter was placed into a femoral vein for injection of supplemental anesthesia (10–30 mg/kg; as necessary). A femoral artery was cannulated for the measurement of arterial blood pressure and to obtain a blood sample for the determination of blood gases.

In all studies, a craniotomy was prepared over the left parietal cortex to visualize the cerebral microcirculation. The microcirculation was suffused with a bicarbonate buffer (2 ml/min) that was bubbled continuously with 95% nitrogen and 5% carbon dioxide. Temperature of the suffusate was maintained at 37 ± 1°C. The cranial window was connected via a three‐way valve to an infusion pump, which allowed for the infusion of agonists into the suffusate. This method maintained a constant temperature, pH, pCO2, and pO2 of the suffusate during infusion of drugs. Arterial blood gases were monitored and maintained within normal limits throughout the experimental period. Diameter of cerebral arterioles was measured using a video image‐shearing device.

Responses of cerebral arterioles were examined in male and female rats (4–6 and 14–16 weeks of age) during superfusion of vasoconstrictor agents: arginine vasopressin (AVP; 1 and 10 nM) (Cat. No. 1711100, USP), angiotensin II (1 and 10 µM) (Cat. No. A9525, Sigma), and the thromboxane analog U‐46619 (10 and 100 nM) (Cat. No. 16450, Cayman Chemical). Diameter of arterioles was measured before, and at 1‐minute intervals for 5 minutes during the application of agonists. Baseline diameter of cerebral arterioles returned to control levels (before application of agonists) within 2–3 min after application of agonists was stopped. All rats were euthanized at the end of the experiment.

### 2.3 Statistical analysis

We report the percent change in diameter of cerebral arterioles during application of agonists. Analysis of variance (ANOVA) with Fisher's least significant difference (LSD) was used to compare responses of cerebral arterioles to the agonists in male and female control and prenatal alcohol rats. Values are mean ± SEM. A *p*‐value of 0.05 or less was considered to be significant.

## 3 RESULTS

### 3.1 Control parameters

Baseline diameter of cerebral arterioles and mean arterial pressure was similar (*p* > 0.05) in adolescent (4–6 week‐old) and adult (14–16 week‐old) male and female control rats and rats exposed to alcohol in utero (Table 1).

**TABLE 1:** Baseline diameters of cerebral arterioles and mean arterial pressure

	Control		Alcohol	
	Adolescent	Adult	Adolescent	Adult
Diameter (microns)				
Male	46 ± 6 (8)	47 ± 3 (8)	52 ± 7 (7)	40 ± 3 (7)
Female	50 ± 6 (6)	43 ± 2 (7)	42 ± 5 (6)	46 ± 4 (8)
Mean Arterial Pressure (mmHg)				
Male	97 ± 8 (8)	111 ± 5 (8)	99 ± 6 (7)	114 ± 3 (7)
Female	105 ± 5 (6)	112 ± 9 (7)	100 ± 5 (6)	111 ± 11 (8)

Note

Values are mean ± SE. Subject numbers for various groups are shown in parentheses. There were no significant differences in baseline diameter or mean arterial blood pressure between the animals in the various groups.

### 3.2 Response to agonists

**FIGURE 1:**
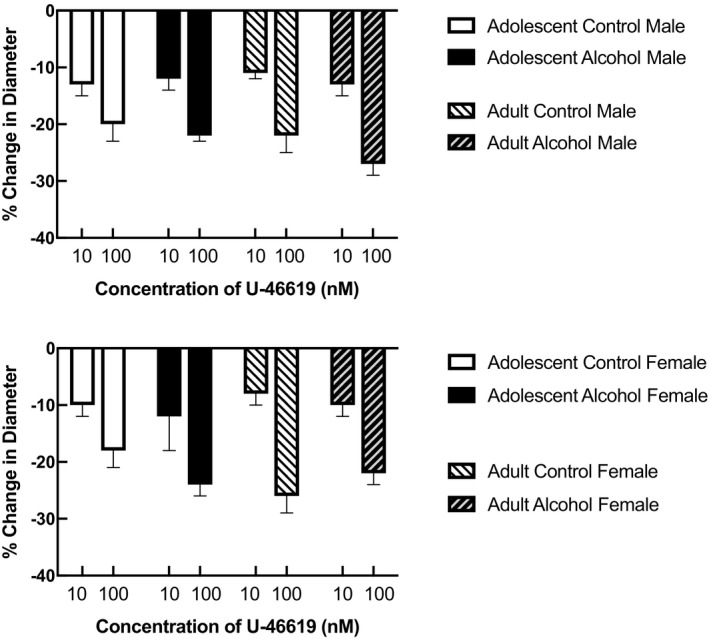
Responses of cerebral arterioles to the thromboxane analog, U‐46619, in adolescent (4–6 weeks old) male and female control rats (*n* = 5 for female and *n* = 6 for male) and rats exposed to prenatal alcohol (*n* = 6 for female and *n* = 5 for male) and adult (14–16 weeks old) male and female control rats (*n* = 7 for female and *n* = 8 for male) and rats exposed to prenatal alcohol (*n* = 6 for female and *n* = 8 for male). Values are means ± SD

Topical application of the thromboxane analog, U‐46619, constricted cerebral arterioles in all groups of rats (Figure 1). There were no differences in the magnitude of vasoconstriction in response to U‐46619 between male and female control rats and rats exposed to prenatal alcohol at either adolescence or adulthood (Figure 1; *p* > 0.05).

**FIGURE 2:**
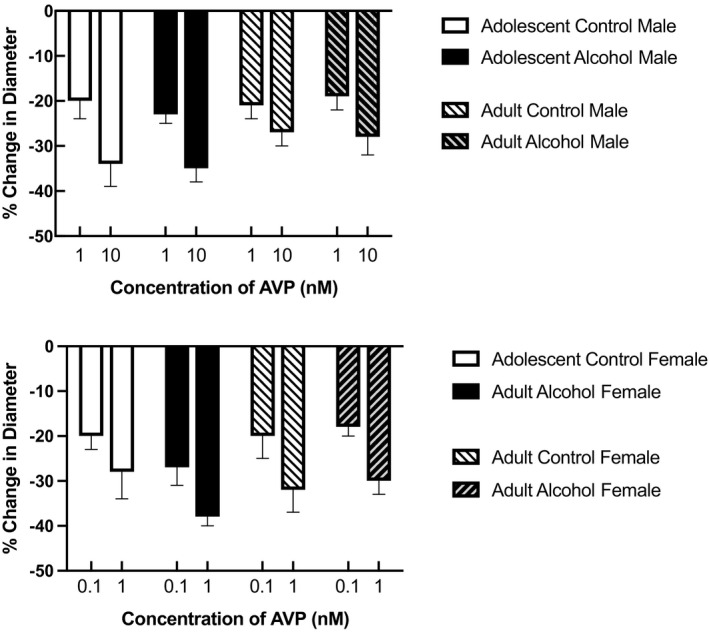
Responses of cerebral arterioles to arginine vasopressin (AVP) in adolescent (4–6 weeks old) male and female control rats (*n* = 6 for female and *n* = 8 for male) and rats exposed to prenatal alcohol (*n* = 6 for female and *n* = 7 for male) and adult (14–16 weeks old) male and female control rats (*n* = 7 for female and *n* = 8 for male) and rats exposed to prenatal alcohol (*n* = 6 for female and *n* = 8 for male). Values are means ± SD

Similarly, AVP constricted cerebral arterioles in all groups of rats (Figure 2). There were no differences in the magnitude of vasoconstriction in response to AVP between male and female control rats and rats exposed to prenatal alcohol at either adolescence or adulthood (Figure 2; *p* > 0.05).

**FIGURE 3:**
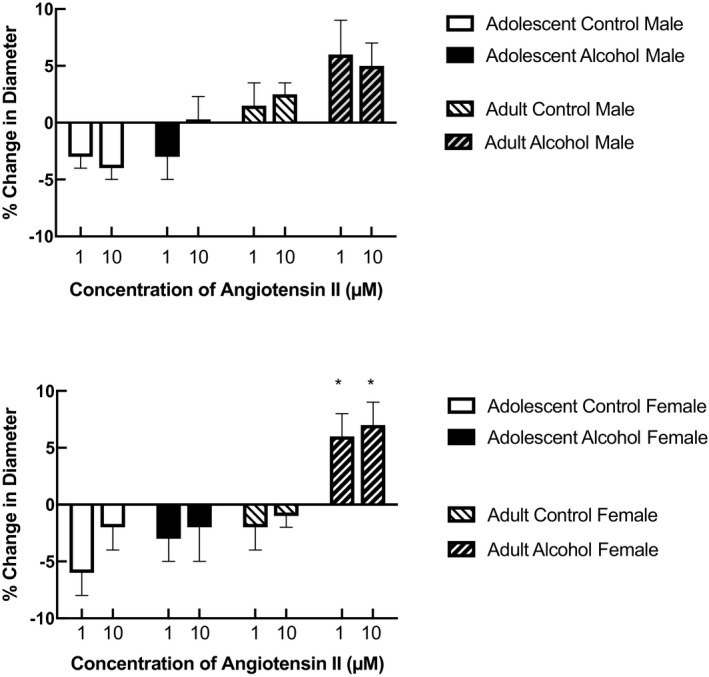
Responses of cerebral arterioles to angiotensin II in adolescent (4–6 weeks old) male and female control rats (*n* = 6 for female and *n* = 8 for male) and rats exposed to prenatal alcohol (*n* = 6 for female and *n* = 7 for male) and adult (14–16 weeks old) male and female control rats (*n* = 5 for female and *n* = 7 for male) and rats exposed to prenatal alcohol (*n* = 7 for female and *n* = 8 for male). Values are means ± SD. *p < 0.05 versus response in control rats

Application of angiotensin II produced minimal changes in the diameter of cerebral arterioles in adolescent male and female control rats, which were equivalent to that seen in rats we exposed to prenatal alcohol (Figure 3; *p* > 0.05). Similarly, responses to angiotensin II were not altered between adult male control and prenatal alcohol rats (Figure 3; *p* > 0.05). In contrast, there was a significant difference in responses of cerebral arterioles to the highest concentration of angiotensin II (10 µM) in adult female rats exposed to prenatal alcohol when compared to their control counterparts (Figure 3; *p* < 0.05). In these rats, responses reversed from a small vasoconstriction to a modest vasodilation to the high concentration of angiotensin II.

## 4 DISCUSSION

This is the first study to examine in vivo responses of cerebral resistance arterioles to vasoconstrictor agents in rats exposed to prenatal alcohol. There are two findings from this study. First, it does not appear that prenatal exposure to alcohol alters responses of cerebral arterioles to thromboxane and AVP in male and female adolescent and adult rats. Second, it appears that prenatal exposure to alcohol does not influence responses of cerebral arterioles to angiotensin II in adolescent male or female rats, or in adult male rats. However, responses to a high concentration of angiotensin II were reversed from vasoconstriction to vasodilation in adult female rats exposed to prenatal alcohol. Taken together, our findings appear to suggest that prenatal exposure to alcohol does not dramatically alter constrictor responses of cerebral arterioles.

### 4.1 Response to the agonists

Our rationale for examining responses of cerebral arterioles to the thromboxane analog (U‐46619) is based upon our previous study in which we found that dilator responses of cerebral arterioles to another product released by platelets, that is, ADP, is impaired in rats exposed to prenatal alcohol (Cananzi & Mayhan, 2019a). Thus, we reasoned that constrictor responses to thromboxane may also be influenced by prenatal exposure to alcohol. ADP appears to dilate cerebral arterioles via the synthesis/release of nitric oxide, however, the role of the endothelium in modulating responses to thromboxane (U‐46619) is mixed. Some have reported that contraction of canine or porcine coronary arteries to U‐46619 is similar in endothelium‐intact and endothelium‐denuded preparations (Cocks & Angus, 1983). Others have shown that contraction of coronary arteries from rabbits and the aorta from rats is similar in endothelium‐intact and endothelium‐denuded preparations (Bullock et al., 1986; Kanmura et al., 1987). We have reported that constriction of cerebral arteries to U‐46619 was similar before and after inhibition of nitric oxide synthase with L‐NMMA (Mayhan, 1998). Thus, it does not appear that nitric oxide contributes to constriction of cerebral arterioles in response to U‐46619. Although many studies have examined the influence of disease states on reactivity of blood vessels to thromboxane, no studies that we know have examined the influence of prenatal exposure to alcohol on the reactivity of cerebral arterioles to thromboxane. In the present study, we found that constrictor responses of cerebral arterioles to U‐46619 were not altered in adolescent and adult male and female rats that were exposed to prenatal alcohol. Thus, in contrast to that reported for ADP, it does not appear that prenatal exposure to alcohol alters responses of cerebral arterioles to thromboxane.

Several studies have suggested that prenatal exposure to alcohol can influence the AVP axis in the brain. Rojas‐Castañeda et al. (2000) found morphological changes in AVP‐producing cells of the suprachiasmatic nucleus in rats exposed to prenatal alcohol. Lee et al. (Lee et al., 2000) reported a reduction in AVP mRNA in the parvocellular region of the paraventricular nucleus in rats exposed to prenatal alcohol. In addition, Bird et al. (2006) report a blunted AVP release in response to hemorrhage in adult male rats exposed to prenatal alcohol. This finding suggests an alteration in the reflex control of blood pressure at a time of reduced blood volume in animals exposed to prenatal alcohol, possibly related to a decrease in vasopressinergic neurons, a decrease in vasopressin receptors and/or a decrease in the synthesis/release of AVP in the brain. These morphological and functional changes to AVP‐producing cells are thought to contribute to many neuropsychiatric disorders associated with prenatal exposure to alcohol (Harper et al., 2018). Based upon these previous studies, we reasoned that altered responses of cerebral arterioles to AVP, leading to impaired regulation of cerebral blood flow, may contribute to some of these abnormalities associated with prenatal exposure to alcohol. However, we found that constrictor responses of cerebral arterioles were similar in male and female control rats and rats exposed to prenatal alcohol at two different ages (adolescent and adult). This finding suggests that prenatal exposure to alcohol probably does not lead to alterations in AVP receptors on cerebral resistance arterioles to influence the regulation of cerebral blood flow. Therefore, changes to AVP‐mediated arteriolar responses most likely do not contribute to morphological, functional, and behavioral changes observed by prenatal exposure to alcohol.

We chose to examine the role of angiotensin II on vascular function given that tissue and circulating levels of angiotensin II, and activation of angiotensin II type 1a (AT‐1a) receptors are increased by alcohol consumption in adult animals and humans (Ibsen et al., 1981; Matyas et al., 2016; Wright et al., 1986). So, although there are no studies that we know of that have examined levels of angiotensin II during exposure to prenatal alcohol, we suggest that altered vascular function in response to angiotensin II may have implications to the regulation of cerebral blood flow. We found that angiotensin II produced minimal changes in the diameter of cerebral arterioles in adolescent male and female rats regardless of exposure to prenatal alcohol. However, in adulthood, angiotensin II produced a small, but significant dilation of cerebral arterioles in rats exposed to prenatal alcohol that was only evident in females. We and others have shown that activation of AT‐1a receptors with angiotensin II produces dilation of cerebral arterioles that is, related to an increase in oxidative stress via NADPH oxidase (Arrick et al., 2008; Chrissobolis et al., 2012; De Silva & Faraci, 2012; Zimmerman et al., 2004). In addition, we have shown that the capacity to produce superoxide, when stimulated with NADPH, is increased in adult rats exposed to prenatal alcohol (Cananzi & Mayhan, 2019b). However, this increase in superoxide production during stimulation with NADPH was similar in brain tissue from male and female adult rats exposed to prenatal alcohol. We suggest that it is possible that stimulation with angiotensin II would produce greater increases in superoxide production from brain tissue obtained from adult female rats exposed to prenatal alcohol when compared to the other groups of rats (control and alcohol exposed), but we have not examined this possibility. Further, the mechanism for this proposed increase in capacity to produce oxidants in response to angiotensin II in female rats exposed to prenatal alcohol is not clear from the present studies.

In summary, this is the first study to examine the influence of prenatal exposure to alcohol on constrictor responses of cerebral arterioles. We found that prenatal exposure to alcohol did not alter vasoconstriction to the thromboxane analog, U‐46619, or AVP in adolescent and adult male and female rats. We also found that responses to angiotensin II were not altered by exposure to alcohol in adolescent male and female rats. However, responses of cerebral arterioles to the high concentration of angiotensin II in adult female rats were altered as a function of prenatal alcohol, reversing from a small constriction to a modest dilation. Given that we have shown in previous studies that NOS‐dependent dilation of cerebral arterioles are altered by prenatal exposure to alcohol, we suggest that impaired dilation of cerebral arterioles, coupled with preservation of vasoconstriction, may contribute to an increase in the susceptibility of the brain to damage during periods of ischemia.

## CONFLICT OF INTEREST

None.

## AUTHOR CONTRIBUTION

All authors contributed to the work presented in this paper. WGM and DMA conceived the study; PSS, DMA, TMK, MW, JS, and WGM were involved in the design the experiments, performing the experiments, analyzing the data, writing of the manuscript, and reviewing the manuscript prior to submission.
